# A rare case of a giant cystic leiomyoma presenting as a retroperitoneal mass

**Published:** 2014-12

**Authors:** Ümit Naykı, Cenk Naykı, Paşa Uluğ, Ismayil Yılmaz, Zeliha Cetin, Yusuf Yıldırım

**Affiliations:** 1 *Department of Obstetrics and Gynecology, Erzincan University, School of Medicine, Erzincan, Turkey. *; 2 *Department of General Surgery, Erzincan University, School of Medicine, Erzincan, Turkey.*; 3 *Department of Pathology, Erzincan University, School of Medicine, Erzincan, Turkey.*

**Keywords:** *Uterine leiomyoma*, *Giant Leimyoma*, *Retroperitoneal mass*, *Cystic degeneration*

## Abstract

**Background:**

Giant retroperitoneal uterine leiomyomas are uncommon. Degenerative changes of a leiomyoma may lead to unusual presentation resulting in misdiagnosis preoperatively. The final diagnosis can be made either intraoperatively or histologically.

**Case:**

We report a 45-year-old multiparous women presented with abdominal distension and fatigue for six months. Abdominopelvic Sonography and computed tomography showed a large cystic mass that filled the pelvis and abdomen. With the preoperative diagnosis of a malignant tumor, a laparotomy was planned. Intraoperatively, a cystic mass originated from the uterus near the left side of the broad ligament extending to the retroperitoneal space was observed. Total hysterectomy and bilateral salphingo-oopherectomy was administered. The histology revealed a leiomyoma with cystic degeneration.

**Conclusion:**

Retroperitoneal leiomyomas should be kept in mind in the diferrential diagnosis of a giant cystic mass in abdomen.

## Introduction

Leiomyomas are common bening tumors in females of reproductive age that arise from uterine smooth muscle (1). The size of leiomyomas varies from microscopic to giant. While most often straightforward in their presentation and management, they can undergo various kinds of asymptomatic degeneration that makes the diagnosis difficult. However, giant retroperitonal leiomyomas are rare in the general practice of gynecology and this rarity makes it an unexpected incident that is either mistaken preoperatively for a retroperitoneal mass (2, 3). We present case of a giant cystic intraligamentary leiomyoma presenting as a retroperitoneal mass.

## Case report

A 45-year-old, multiparous woman admitted to Erzincan University Hospital in 2013 with a history of abdominal distension and fatigue for six months. Her menstruel cycle was regular. She had no history of a serious illness and surgery. Physical examination revealed a huge semi-mobil abdominal mass that caused distension. No abdominal tenderness was present. Uterus and bilateral adnexes could not be palpated because of the mass. Vital signs were normal. An abdominal sonogram revealed a large cystic mass with multiple septations occupying the whole abdomen. It was difficult to understand the origin of the mass and its nature. Uterus and endometrium was normal, but bilateral ovaries could not be detected. 

**Figure 1:**
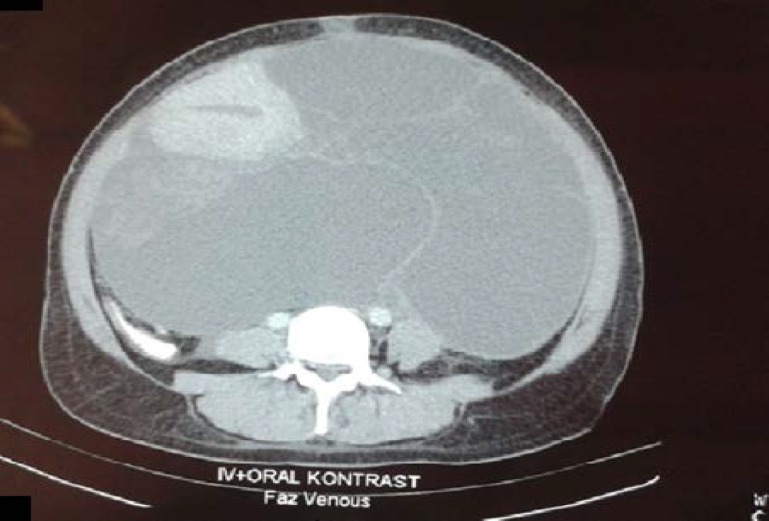
Computered Tomography (CT) of the giant retroperitoneal leiomyoma with cystic degeneration and a normal uterus

An abdominopelvic computed tomography (CT) showed a huge cystic mass with multiple septations, approximately 30x26x17 cm in size, occupying the abdomen from pelvis to the upper abdominal cavity. The tumor superiorly deplased the bladder and uterus, laterally compressed the small intestine (Figure 1). The laboratory results including serum electrolyte levels, tests of liver and renal functions were normal. However, haemoglobin was 6.79 g/dl and haematoctrit was 23.6%. Tumor markers (CA-125, CA19-9, CA 15-3, CEA and AFP) were also within normal limits. The patient was informed and was taken written approval for the operation and the report.

After four units of blood transfusion, laparotomy was attempted. An abdominal midline xiphopubic vertical insicion was made. At laparotomy, we observed a large, multilobated and predominantly cystic retroperitoneal tumor that originated from broad ligament and extended to the xyphoid with occupying the whole pelvis. Adhesions between the mass and posterior of the transverse colon were noted. Uterus was in normal size but displaced up in the right of the pelvis and bilateral ovaries were normal. 

**Figure 2:**
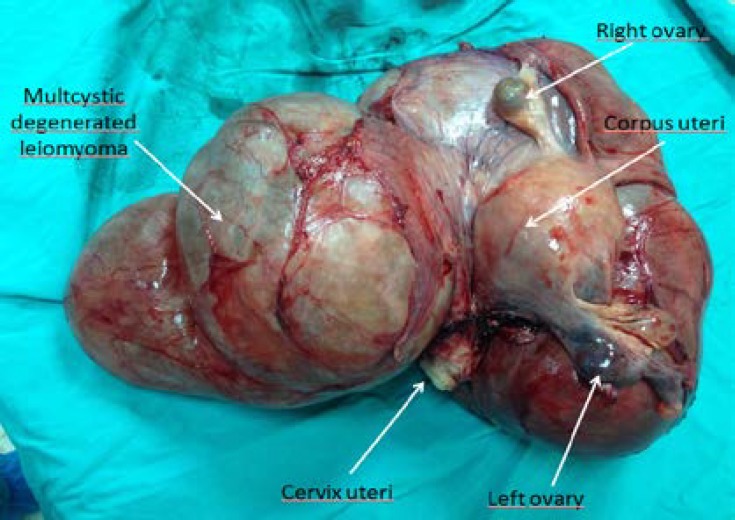
Surgical material of the uterus, bilateral ovaries and giant intraligamentary cystic mass

A total abdominal hysterectomy and bilateral salphingo-oopherectomy was performed after en-bloc resection of the tumor. There was no serious bleeding. A drain was packed into the pelvis after obtaining hemostasis. The drain was removed on the third post-operative day and the patient was discharged 10 days after the operation in excellent condition. The resected periadnexial mass that was adjacent to the posterior of the uterus (14x8x5 cm) and normal ovaries (Figure 2), was respectively 36x28x20 cm in size and weighed 3000gr. It was composed of solid and mostly cystic components. 

**Figure 3:**
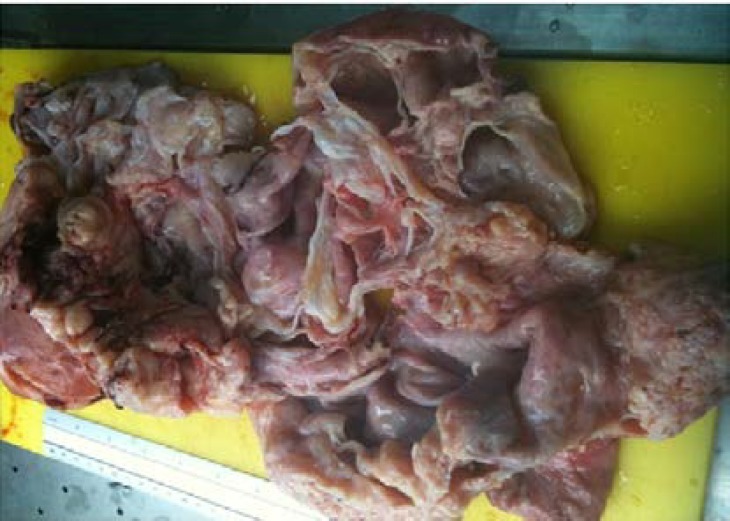
Macroscopic pathology of the multilobulated cystic leiomyoma

The cystic portions contained serous fluid. Microscopic examination revealed a multilobulated leiomyoma with cystic degenerative changes. Histopathologically, it was observed that the cystic areas were surrounded by the smooth muscle cells (Figure 3). Histological signs of malignancy were not found. The final diagnosis which was corrected by the immunohistochemical staining was a giant uterine leiomyoma with marked cystic degeneration occupying the retroperitoneal space. 

**Figure 4:**
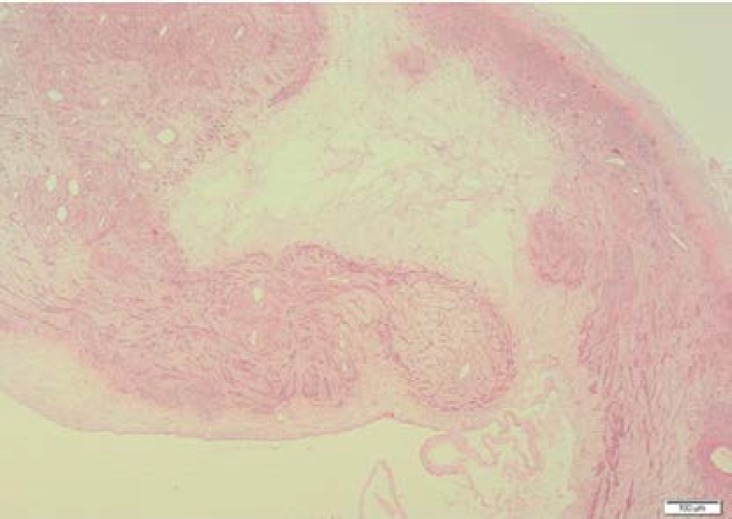
Microscopic findings of the giant reroperitoneal leomyoma with cystic degeneration. Cystic spaces surrounded by smooth muscle cells are seen (Hematoxylin and eosin x20).

## Discussion

Uterine leiomyomas are highly prevalent bening tumors affecting 25% of women in reproductive age (2). Traditionally, leiomyomas grow in the uterus and they are classified as submucosal, intramural or subserosal based on their localization (3). They can be asymptomatic or can cause variety of symptoms, including abnormal bleeding, pain, infertility, miscarriage and tumour bulk-related symptoms. However, some unusual extra uterine growth presentations are mentioned in the literature; bening metastasizing leiomyoma, disseminated peritoneal leiomyomatosis, intravenous leiomyomatosis, parasitic leiomyoma and retroperitoneal growth (4). Retroperitoneal tumors are rare and most are malignant (5). 

The diagnosis of a bening retroperitoneal tumor includes leiomyoma, mature teratoma, schwannoma, lipoma, lympangioma, and neurofibroma. The incidence of leiomyoma among them is 0.5-1.2% (6, 7). Subserosal leiomyomas sometimes grow lateraly between the folds of broad ligament and then extend into the retroperitoneum (8, 9). As these tumors are relatively asymptomatic; they may become very large before the patient becomes aware of them. Of the reported retroperitoneal leiomyomas, 73% of them are located in the pelvis (6). As leiomyomas enlarge, they can outgrow their blood supply and demonstrate various types of degeneration, such as, hyaline, cystic, myxoid or red degeneration, depending on their location or size (10, 11). 

Cystic degeneration, observed in approximately 4% of leiomyomas, may be considered extreme sequelae of edema (12). However, 11.8% of cystic degeneration has been reported in uterine leiomyomas extending to the retroperitoneal space (6). There are some theories regarding the origin of retroperitoneal leiomyomas such as a parasitic origin or an iatrogenic origin (4, 13). On the other hand, Müllerian cell rests or smooth muscle cells in the retroperitoneal vessels wall have been suggested for the origin of such growths (14). About 40% of patients with such tumors have been reported to have a concurrent uterine leiomyoma or a remote history of hysterectomy for the treatment of a uterine leiomyoma (7). 

However, in our case, the patient had no previous abdominal operation and a concurrent uterine leiomyoma. More investigations are needed to identify the exact etiology. Generally, a uterine leiomyoma has a typical appearance on imaging. However, the atypical appearances followed by degenerative changes can cause confusion in diagnosis. When uterine leiomyomas with cystic degeneration show retroperitoneal growth, it is difficult to diagnose them preoperatively with imaging techniques like USG, CT, and MRI. In most of the published case reports, these tumors were clinically diagnosed as retroperitoneal growths with the suspicion of adnexal malignancy without suspecting leiomyoma (5, 14-17). 

Preoperative diagnosis of a retroperitoneal leiomyoma with cystic degeneration can be difficult because of the rarity, and uncommon sonographic appearance of this tumor. We presented an unusual case of a giant cystic leiomyoma that originated from the uterus near the right side of the broad ligament extending to the retroperitoneal space. Leiomyomas should be considered in the differential diagnosis of a cystic retroperitoneal mass.

## Conflict of interest

There is no conflict of interests of each author.
